# Preferential Allele Expression Analysis Identifies Shared Germline and Somatic Driver Genes in Advanced Ovarian Cancer

**DOI:** 10.1371/journal.pgen.1005755

**Published:** 2016-01-06

**Authors:** Najeeb M. Halabi, Alejandra Martinez, Halema Al-Farsi, Eliane Mery, Laurence Puydenus, Pascal Pujol, Hanif G. Khalak, Cameron McLurcan, Gwenael Ferron, Denis Querleu, Iman Al-Azwani, Eman Al-Dous, Yasmin A. Mohamoud, Joel A. Malek, Arash Rafii

**Affiliations:** 1 Department of Genetic Medicine, Weill-Cornell Medical College, New York, United States of America; 2 Surgery Department, Institute Claudius Regaud, Toulouse, France; 3 Pathology Department, Institute Claudius Regaud, Toulouse, France; 4 Oncogenetics, Centre Hospitalier Regional Universitaire de Montpellier, Montpellier, France; 5 Advanced Computing, Weill-Cornell Medical College in Qatar, Doha, Qatar; 6 Biosciences Department, University of Birmingham, Birmingham, United Kingdom; 7 Genomics Core, Weill-Cornell Medical in Qatar, Doha, Qatar; 8 Stem Cells and Microenvironment Laboratory, Weill-Cornell Medical College in Qatar, Doha, Qatar; University of Washington, UNITED STATES

## Abstract

Identifying genes where a variant allele is preferentially expressed in tumors could lead to a better understanding of cancer biology and optimization of targeted therapy. However, tumor sample heterogeneity complicates standard approaches for detecting preferential allele expression. We therefore developed a novel approach combining genome and transcriptome sequencing data from the same sample that corrects for sample heterogeneity and identifies significant preferentially expressed alleles. We applied this analysis to epithelial ovarian cancer samples consisting of matched primary ovary and peritoneum and lymph node metastasis. We find that preferentially expressed variant alleles include germline and somatic variants, are shared at a relatively high frequency between patients, and are in gene networks known to be involved in cancer processes. Analysis at a patient level identifies patient-specific preferentially expressed alleles in genes that are targets for known drugs. Analysis at a site level identifies patterns of site specific preferential allele expression with similar pathways being impacted in the primary and metastasis sites. We conclude that genes with preferentially expressed variant alleles can act as cancer drivers and that targeting those genes could lead to new therapeutic strategies.

## Author Summary

Identifying genes that contribute to cancer biology is complicated partly because cancers can have dozens of somatic mutations and thousands of germline variants. Somatic mutations are gene variants that arise after conception in an organism while germline variants are gene variants present at conception in an organism. Most methods to identify cancer drivers have focused on determining somatic mutations. In this study we attempt to identify, from a tumor sample, important germline and somatic variants by determining if a variant is expressed (made into RNA) more than expected from the amount of the variant in the genome. The preferred expression of a variant could benefit cancer cells. When applying our analysis to ovarian cancer samples we found that despite the apparent heterogeneity, different patients frequently share the same genes with preferentially expressed variants. These genes in many cases are known to affect cancer processes such as DNA repair, cell adhesion and cell signaling and are targetable with known drugs. We therefore conclude that our analysis can identify germline and somatic gene variants that contribute to cancer biology and can potentially guide individualized therapies.

## Introduction

Identifying genes contributing to tumor biology (driver genes) underlies the design of targeted therapies. The advent of large-scale tumor sequencing in 2006 [1] followed by integrated multi-dimensional TCGA studies [2] brought a wealth of molecular data in different cancers at the somatic mutation, gene expression and copy number variation levels. One surprising result has been the observation that in most studied cancers, there are large differences in somatic mutations in patients. For example, in the case of the TCGA ovarian cancer study [3], there were ~10,000 somatic mutations among 316 patients with only *TP53* found mutated in the majority (96%) of patients. Every other gene was found to be mutated at low frequencies. This heterogeneity was also seen in more recent multi-site ovarian cancer studies [4, 5]. Contrary to the heterogeneity observed at the somatic mutation level, gene expression profiles are more homogeneous with distinct gene expression clusters observed both in the TCGA study and other studies [5, 6].

While both somatic mutation and gene expression studies have yielded large insights into tumor biology, they have several limitations in uncovering driver genes. Somatic mutations do not identify germline variants that contribute to tumor biology, require large patient cohorts, make assumptions about the background mutation rate and have turned out be very heterogeneous. Gene expression array studies, though they uncover sets of genes that correlate with prognosis, do not inform about significant or causative genes and do not indicate whether a mutated form of the gene is being expressed.

One approach to address some of these limitations is to identify preferentially expressed variants of gene. If a specific variant is expressed and if that expression is at a significantly greater or lower level than expected, then this could indicate selection for or against that variant and imply that the gene is playing an important role in the tumor. This approach, called previously allelic expression bias analysis [7–9], typically determines significance if the expression allele fraction deviates from 0.5 (the expected non-biased allele expression at heterozygous positions). Allelic expression bias analysis however is difficult to apply to patient tumor samples because samples are often a mixture of normal and tumor cells with tumor cells themselves being heterogeneous with large copy number changes. Therefore, assumptions that an allele has biased expression if it differs significantly from a specific allele fraction are not justified.

However, combined genome and transcriptome sequencing (CGTS) as described here makes it possible to directly assess the genomic content of the sample at all sites and therefore determine if allelic expression is significantly different than expected from the genome content. A resulting advantage of CGTS is also a large reduction of alignment bias [10].

We applied CGTS to primary and metastatic epithelial ovarian cancer samples. Ovarian cancer is the most aggressive gynecological malignancy in developed countries. Ovarian cancer had been thought to arise from ovarian epithelial cells but more recent studies [11–15] have shown that at least 50% of ovarian tumors most likely arise from the fallopian tube. Regardless of origin, the ovaries and abdominal cavity are mostly impacted with more than 70% of patients diagnosed with disease spread throughout the abdominal cavity. These patients have a 30% five year survival rate [16]. Ovarian cancer dissemination most commonly occurs through the intraperitoneal route, followed by lymphatic invasion [17–19]. While 80% of patients with advanced epithelial ovarian cancer initially respond to primary treatment, most recur with a drug resistant phenotype. A subset of patients with clinically and pathologically indistinguishable disease develops a less aggressive disease and may survive much longer. Consequently, patients have biologically different diseases [20]. Furthermore, disease in the same patient can be biologically different according to tumor location and according to tumor temporal variations [21, 22]. Intratumor heterogeneity within the same patient is clinically relevant because status of predictive biomarkers could be used to adapt treatment decisions [23].

Given that patients mostly present with metastasis and that lethality is high at this stage, we include in our study matched primary and metastasis samples from three ovarian cancer patients. The primary sample is from the ovary and the metastasis samples are from the peritoneum and lymph node, the two most frequent metastasis sites [24]. We identify a biologically interesting set of shared significant preferentially expressed alleles, predict patient-specific drug targets and identify site-specific genes that may contribute to specific biology. These results suggest that preferentially expressed variant analysis can identify potential cancer drivers.

## Results

### Identifying Significant Preferentially Expressed Alleles

**Fig 1:**
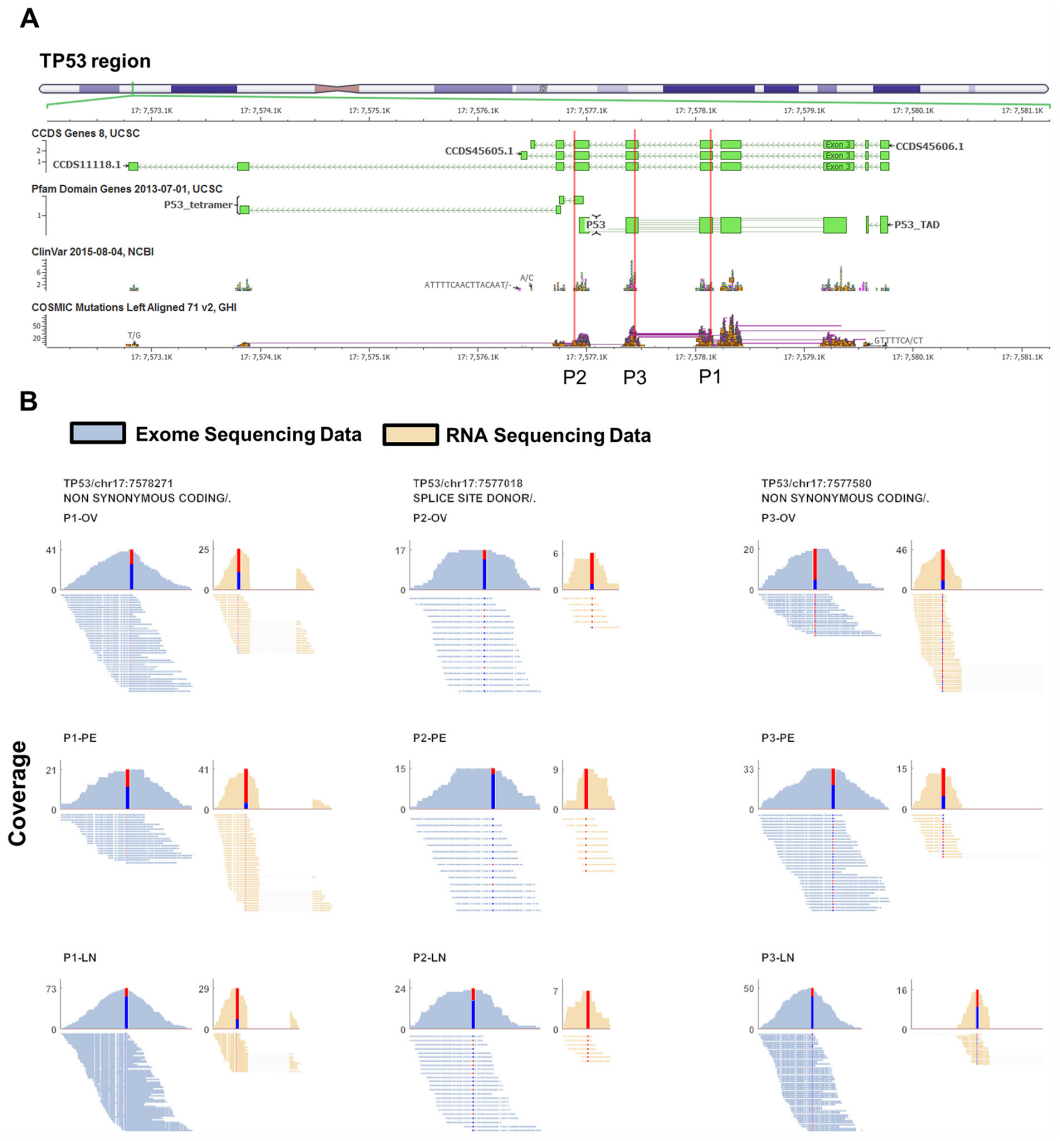
TP53 significant preferential allele expression. We observed in three different patients (P1, P2, P3) alleles in TP53 where one allele is significantly preferentially expressed. A) The allele positions are shown on a schematic view of the TP53 gene (red line) for all three patients. B) Alignment and coverage plots of the reads spanning the preferentially expressed alleles for both exome (blue) and RNA (brown) sequencing data. The patient and site is indicated on each plot. The histograms indicate coverage across the site with the coverage indicated by the y-axis number. The histogram central stacked bar plot shows the number of reference allele reads in blue and the alternate allele reads in red. The alignment plot shows the individual reads spanning the preferentially expressed alleles. For the RNA sequencing data, the dashed line indicate gaps corresponding to a spliced region.

Our approach to identify causative mutations using both RNA and exome sequencing data was motivated by an observation made about the variants present in *TP53* in three patients. In our dataset, each patient had a different high or moderate impact variant in *TP53*. High and moderate impact variants are those changing the protein sequence or that affect a splice site. When comparing the RNA and exome sequencing data of those variants, we observed that while the variant alleles were present in both the RNA and exome data, the TP53 variant allele was over-represented in the RNA sequencing data when compared to the exome sequencing data (Fig 1). Given the known centrality of *TP53* variants in cancers (TP53 somatic mutations have been observed in 96% of ovarian cancer samples [3]), this suggested to us that there could be a selection process occurring in these patients where variant *TP53* is overexpressed or normal *TP53* is suppressed or both.

**Fig 2:**
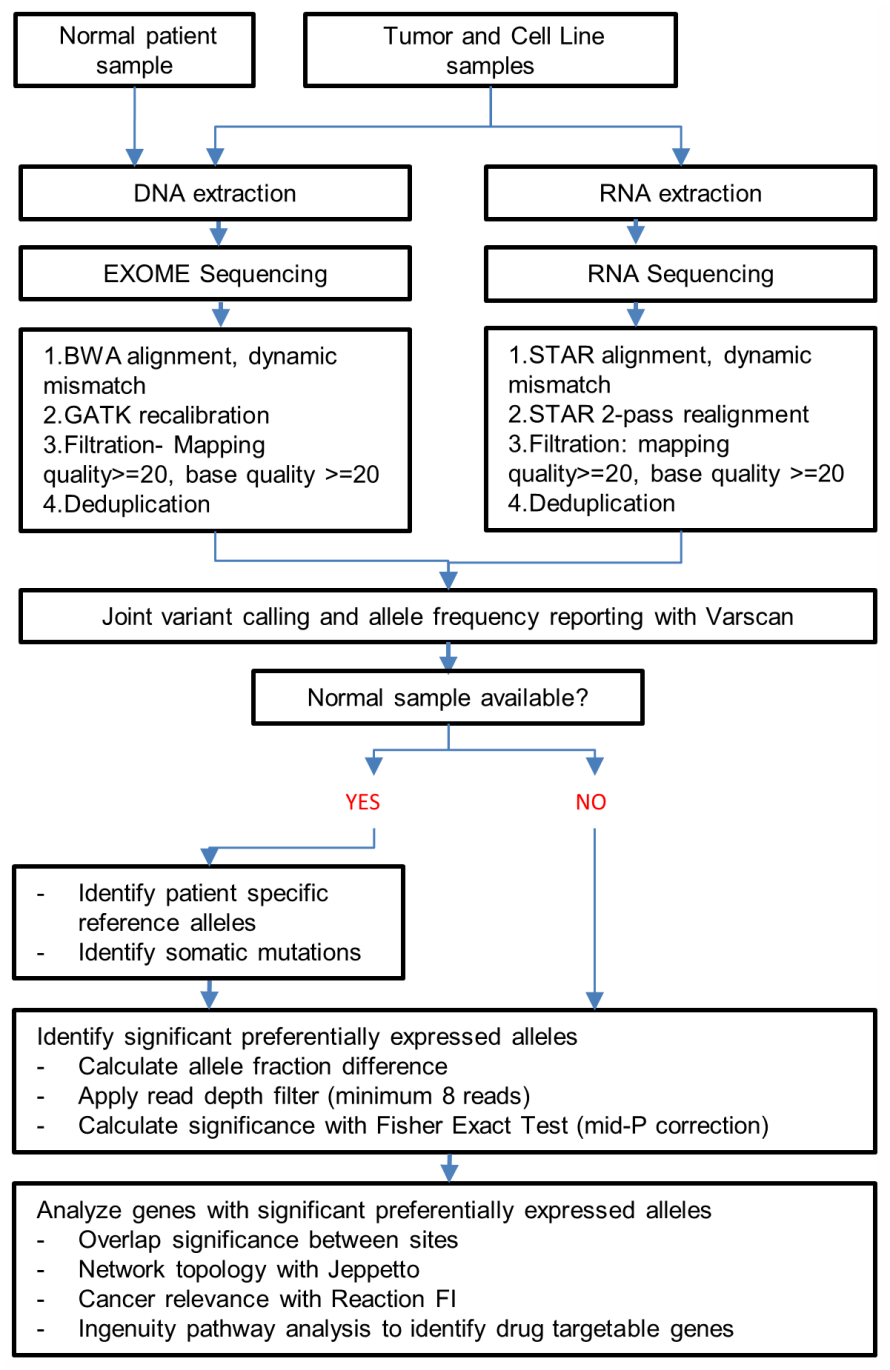
Schematic for global analysis of preferential allelic expression showing the steps followed for alignment, filtration, quality control and analysis steps.

To understand if this preferential allele expression exists in other genes, we performed a systematic analysis at all sites where RNA or exome sequencing data exists as outlined in Fig 2. We performed both an analysis for somatic mutation preferential expression and a combined germline/somatic variant preferential expression. In both analysis, we identify variant positions and calculate the reference allele fraction which is the number of reference allele reads divided by the total number of reads. Using the RNA sequencing data of a sample that correspond to the exome variant position, we also determine the RNA reference allele fraction. Then, we calculate the significance of the difference as described more fully in the methods. Throughout this study, we adopt a convention where the reference allele fraction is the standard and the alternate allele is the variant or mutant allele. Differences between the RNA and exome allele fractions are calculated as RNA reference allele fraction—Exome reference allele fraction (abbreviated as RAD). Therefore, if the RNA reference allele fraction is 0.5 and the exome allele reference allele fraction is 0.9, this means that there is preferential expression of the variant allele with an allele fraction difference (RAD) of -0.4.

**Fig 3:**
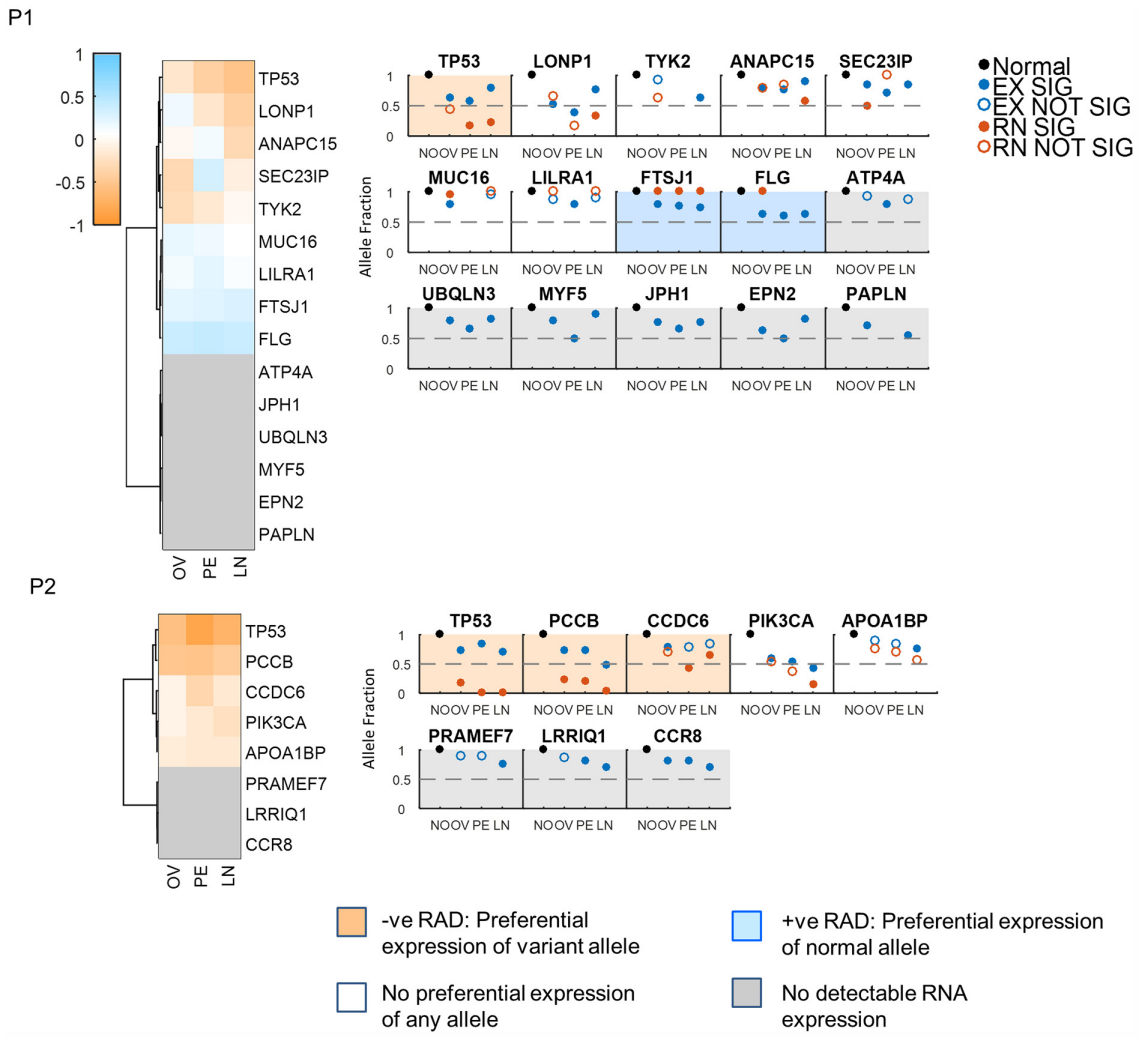
Somatic mutations preferential allele expression in patient 1 (P1) and patient 2 (P2). Clustering of the allele fraction differences for all somatic genes shows somatic mutations with preferential allele expression for the mutant allele (orange), no preferential expression (white), reference allele preferential expression (blue) and no expression of any allele (gray). Subplots for each patient show the exome (blue circles) and RNA (red circles) allele fraction for every site (NO is normal, OV is ovary, PE is peritoneum and LN is lymph node). Filled blue circles indicate significant difference from 1 and filled red circles indicate significant difference from the exome allele fraction for the given sites. Open circles indicate no significant difference. An absence of a circle indicates no data for that site. Plots are sorted from the highest allele fraction difference to the lowest. Genes with no RNA expression were arbitrarily assigned a highly negative allele fraction difference. Shaded plots indicate if there’s significant preferential allele expression and the direction of the expression as stated in the legend. Note that shading was done as an aggregate of all sites within a patient. For example, in P2, one site in PIK3CA has a significant allelic difference (the LN) while there is no difference at other sites.

The analysis of all somatic mutations (in two patients where non-cancer tissue is available) reveals mutated genes where the mutated allele is preferentially expressed (Fig 3). TP53 mutation is found to be significantly preferentially expressed in patient 1 while TP53, PCCB and CCD6 mutations are found to be significantly preferentially expressed in patient 2. In addition, this analysis shows variants that are not expressed, variants that are expressed at equal levels as in the genome and variants where the germline variant is preferentially expressed (Fig 3). These results reveal that an analysis of somatic mutation is better complemented with allelic expression studies which show whether a mutated allele is expressed and the degree and direction of that expression. Mutated alleles that are not expressed likely have no impact on tumor progression.

**Fig 4:**
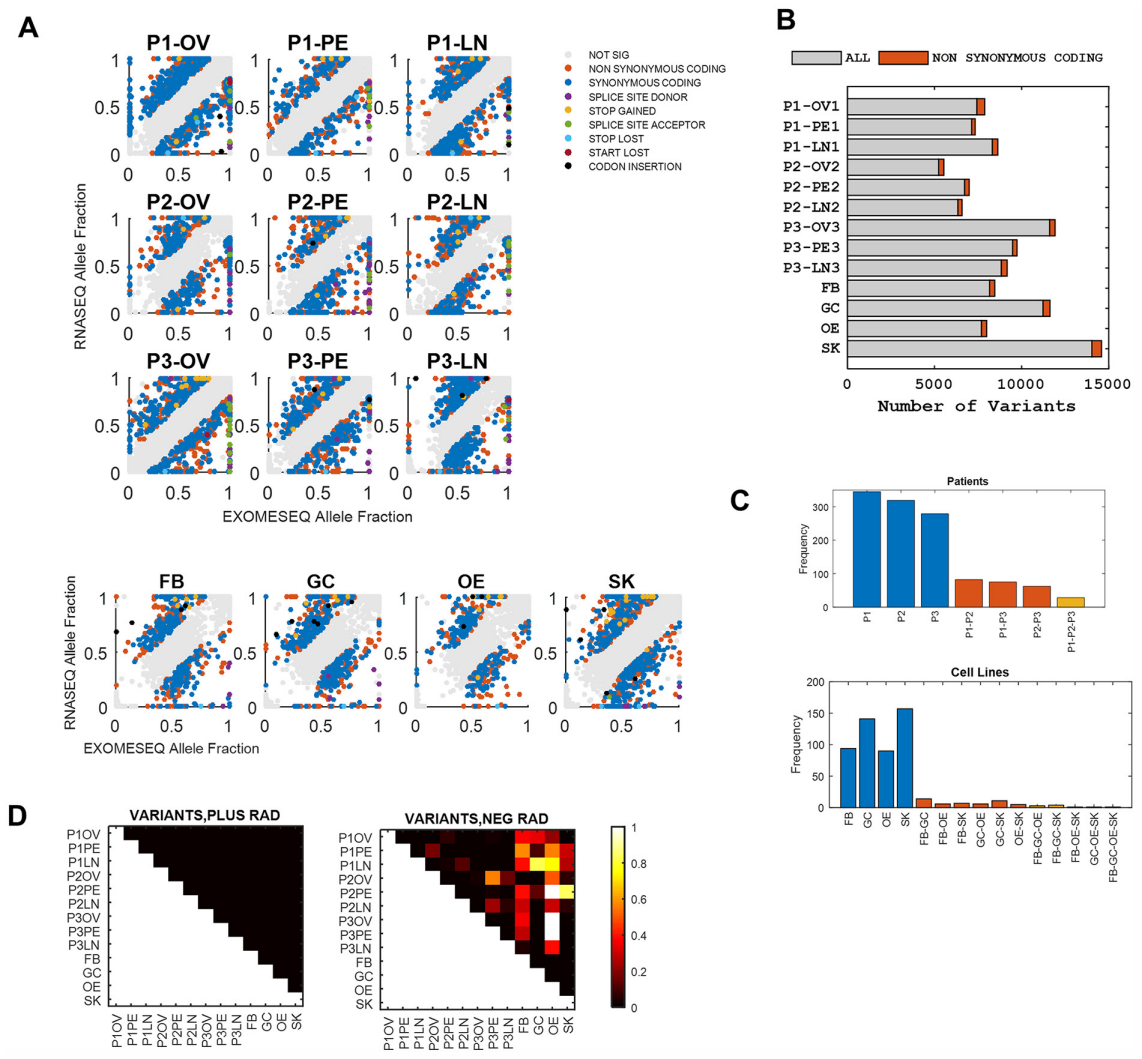
All variant preferential allele expression analysis. A) Scatter plots show the exome and RNA allele fraction for every patient and site in our study and for cells lines (FB, GC, OE, SK). Grey circles are variants with non-significant allelic differences while significant differences are indicated in colored circles as stated in the Legend. Note we imposed a cutoff of allelic difference of 0.2. B) The number of significant non-synonymous coding variants relative to all variants for patients and cell lines. C) The shared variants in patients and cell lines are shown as bar plots. D) Analysis of the significance of shared variants. The approximate p-value for the pair is shown in the heatmap with p-values of 0 being black and p-values of 1 being white. We analyzed the positive RAD variants and the negative RAD variants and found large difference between the cell line data and the patient site.

In addition to analyzing somatic mutations, we analyzed all variant alleles that consist of germline and somatic variants. For most variants, there is no significant difference in the exome and RNA allele fractions (Fig 4A). However, for a relatively small number of variants, there are significant allele fraction differences (Fig 4A and 4B). These variants are both germline and as previously stated somatic with the vast majority (~96%) being germline. Furthermore, using the normal data for two patients we estimated how many reference/alternate alleles would have been incorrectly assigned to be able to estimate the error for the third patient and the cell line data. As S2 Fig shows, the error ranges from 4.5%-9.4%. This means that patient 3 and cell line data likely have up to 10% of their alleles incorrectly called as alternate or reference. This would affect the direction of preferential allele expression in our analysis. However, for our global analysis this error would not significantly affect our major conclusions.

We also performed exome and RNA sequencing on four different cell lines (SKOV3, GOC2, fibroblasts and primary ovarian epithelial cells). As in the cell lines, we identify significant allele fraction differences in a small subset of variants (Fig 4A and 4B). Interestingly, while the same types of variants is observed in both cell lines and patient data, the cell lines show an enrichment in STOP gained variants compared to patient data (S3 Fig).

### Shared Preferentially Expressed Alleles

To analyze the significance of the observed preferentially expressed variants, we compared the cell lines and patient shared genes. We observe a striking difference between cell lines and patient shared genes as the variant genes identified are very different between the two. The patient shared variants are substantial with 28 shared genes while only few genes are shared among the four different cell lines (Fig 4C). No gene is shared between cell lines and patients.

However, the number of significant variants is larger overall in patients which would lead to higher number of shared variants. To determine if the relatively high number of shared variants in patient data is due to chance, we performed a significance analysis. We selected variants randomly from the total pool of variants in the data and counted how many times these variants are shared between patients and cells. We then calculate a p-value corresponding to the number of times the shared random replicates overlaps with the observed shared replicates. As the p-value heatmap in Fig 4D shows, there is significant shared variants among patients and cell lines for the reference variants while there is significant shared variants for the alternate variants only among the patient data. This indicates that the set of shared alternate variants are significant in the context of patient tumors while the set of reference variants are significant for cell lines and patients. We therefore conclude that the set of preferentially expressed alternate alleles are specifically relevant to patient cancer data and focusing on that set could yield to insights into patient tumor biology.

To further understand if the alleles in our data are selected for we carried out a synonymous to nonsynonymous analysis for every patient and site. In this analysis, for every gene with at least 2 variants, we calculate the ratio of the number of non-synonymous to synonymous variants for the positive RAD, negative RAD and no-RAD variants. As shown in S4 Fig, in every patient and site, variants with positive and negative RAD generally have a non-syn/syn ratio greater or equal to the variants with no RAD. This is especially visible at the P1-LN site. This suggests that there could be selection for some of the positive and negative RAD variants. However, this type of analysis is limited in that there were few genes with enough variants to calculate synonymous and non-synonymous ratios as there is little variation within genes from the same patient. For most of the genes, selection cannot be identified using this method.

**Fig 5:**
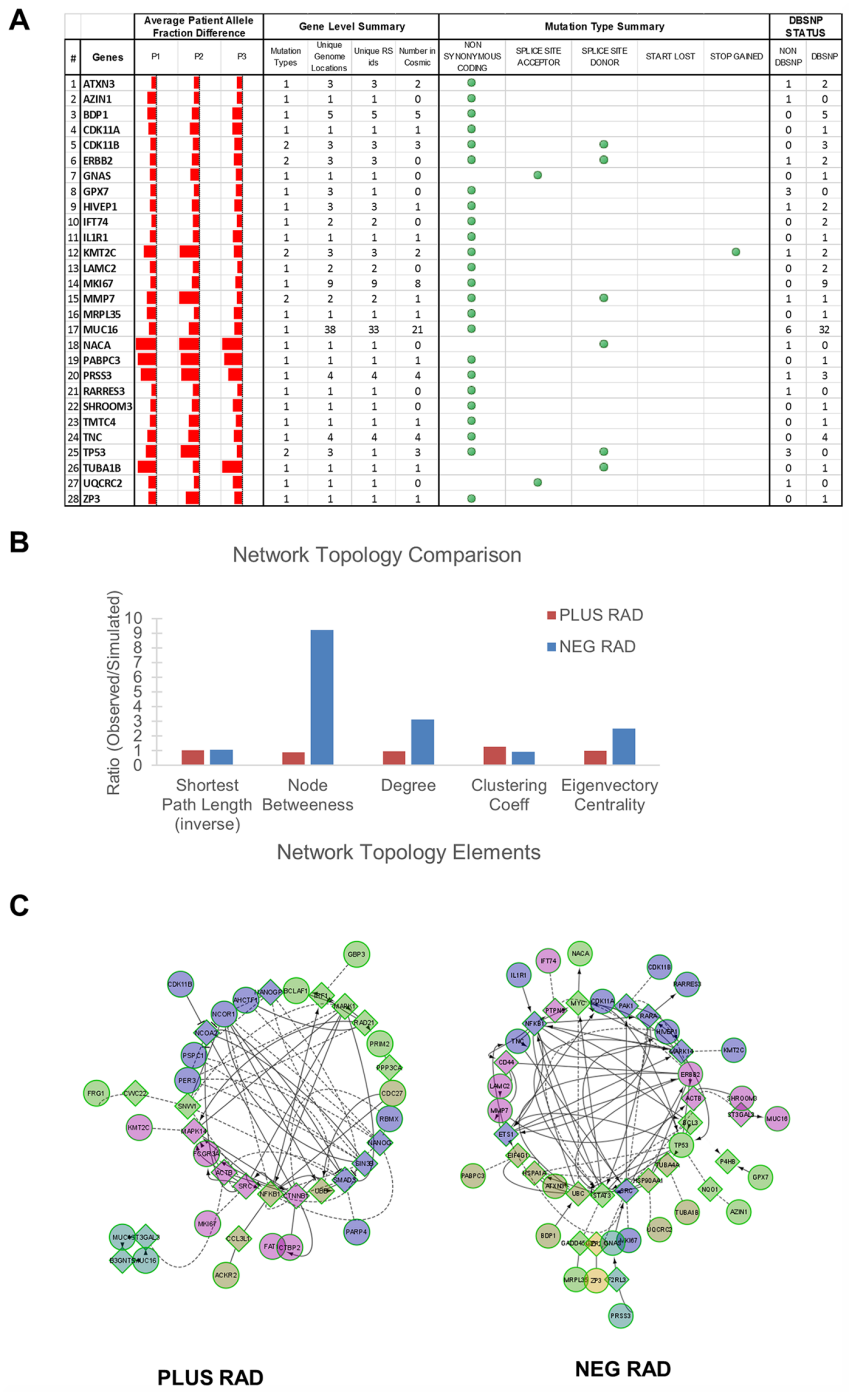
Network analysis of shared patient plus and negative RAD sets. A) List of 28 genes with patient and gene level summaries of the different variants. B) Toplogical analysis of network associations based on known biology among the set of positive and negative RAD genes. C) Association between genes of the negative and positive RAD sets. Each symbol is a gene with colors corresponding to the clustering analysis within that gene. Circle symbols indicate genes shared within our dataset while squares indicate associated genes not in our dataset. Solid lines indicate direct interactions while dotted lines indicate indirect interactions. Note the higher frequency of direct interactions among negative RAD genes.

We next looked into the biological significance of the shared patient’s alternate alleles. There were 28 genes shared across three patients (Figs 4C and 5A). Among the 28 shared genes, the majority are known to be involved in tumor biology based on IPA annotation of the genes and manual inspection. These include *TP53* (the highly mutated in epithelial ovarian cancer tumor suppressor), *MUC16* (also known as CA125) [25–27], *MKI67* [28–31], *LAMC2* [32–34] and ERBB2 [35]. In addition, the corresponding proteins localize to the extracellular space, plasma membrane, cytoplasm and nucleus.

Two genes, *MUC16* and *MKI67*, show a large number of variants (38 and 9 different variants respectively). The large number of variants in these genes could be due to the size of these genes, alignment errors in repeat regions (though we filtered out multi-mapping reads and low quality reads) or naturally polymorphic positions or may be due to heterogeneity in different cells. We include them in the list of significant genes due to their known role in tumor biology and because some of the variants we identified have also been reported as somatic mutations in COSMIC for all two genes (Fig 5A). *MUC16* is known to be expressed in most serous ovarian carcinomas and may function like *MUC1* and *MUC4* in tumor cell growth, motility and tumorogenicity [26]. *MKI67* (Ki-67) is a well-known proliferation marker associating with prognosis [29, 36] and has been used as a target in an ovarian cancer model system [30].

We then performed a topological network analysis using TopoGA [37] and Jepetto [38] on the set of shared 28 genes with alternate allele preferential expression (negative RAD) and the set of shared 26 genes with reference allele preferential expression (positive RAD). As Fig 5B shows, there is a clear increase in connectedness among the alternate allele preferentially expressed genes. Fig 5C also shows a plot of the known genetic interactions from the reactome functional interaction database [39, 40] and there are many more direct interactions for the alternate allele preferentially expressed genes than for the reference allele preferentially expressed genes.

Overall, our results strongly indicate that, in contrast to the set of preferentially expressed reference alleles, the shared set of preferentially expressed alternate/variant alleles play a role in ovarian cancer biology since the majority of the identified genes are involved in known cancer processes.

Additionally, we find that the expression level for most of these 28 genes is remarkably consistent across different patients and sites (S5 Fig). This indicates that these genes with significant allelic bias cannot be identified from gene expression studies.

### Patient and Site Specific Preferentially Expressed Alleles

**Fig 6:**
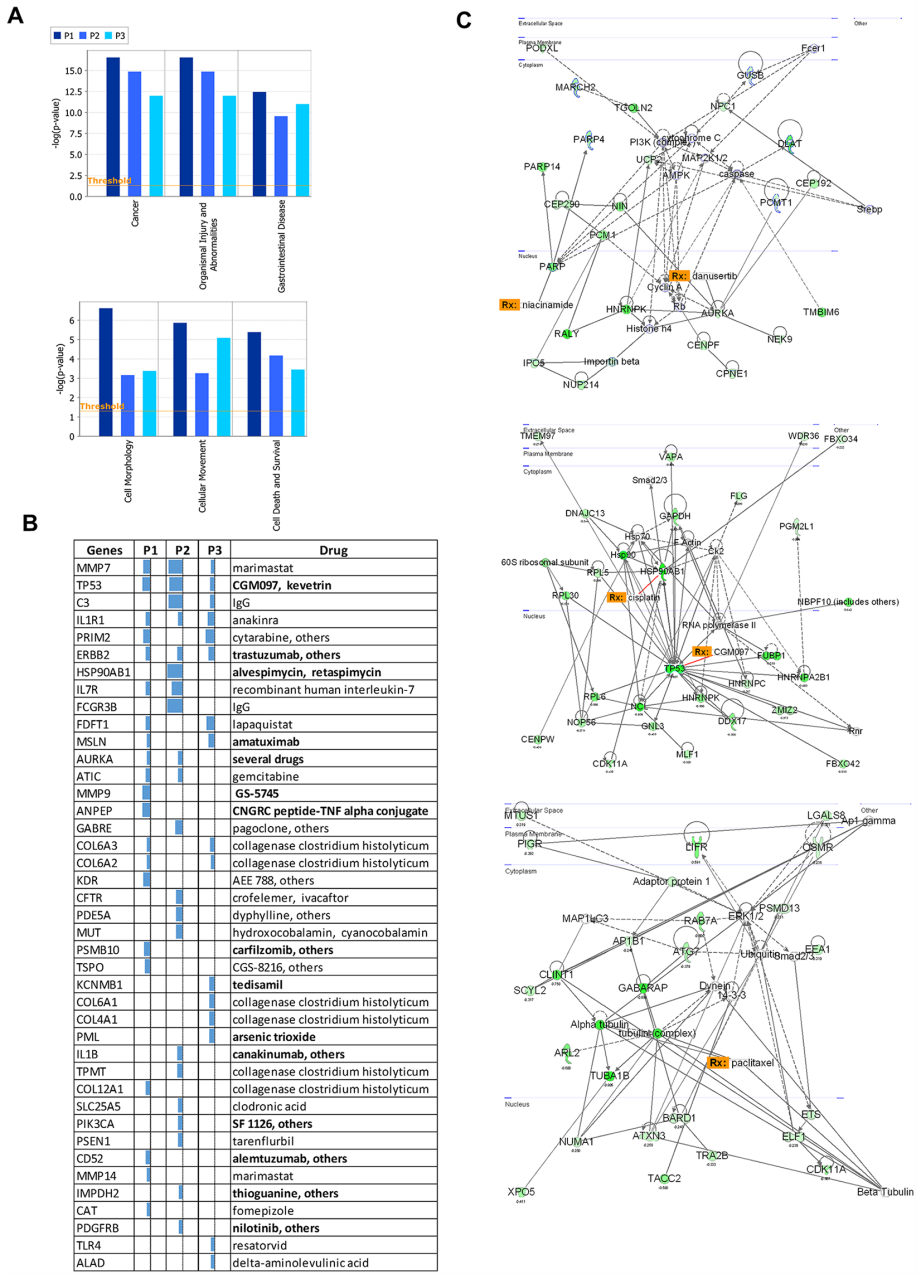
Patient specific analysis of negative RAD genes. A) Top biological pathways molecular functions for every patient’s set of significant preferentially expressed alleles. B) Druggable targets in every patient. The bar plots show the extent of preferential allele expression and the list is sorted by the average of allelic expression from highest to lowest. Drugs in bold type are used in current anticancer treatment or undergoing clinical anticancer trials. C) Network plots of a selected pathway in every patient showing a druggable target.

We then analyzed the genes with preferentially expressed alleles for each patient individually using Ingenuity pathway analysis. Each patient’s significant genes are associated with similar cancer related diseases and processes (Fig 6A). For example for the Cancer category, for patients 1, 2 and 3 at least half of the genes were in cancer associated genes. Note that the gastrointestinal disease reported by IPA include cancer diseases. Similarly there is enrichment for functions such as DNA repair and cell growth for every patient. Our analysis therefore identifies many genes that are known to be involved in tumor development. This is the case whether looking at shared genes or whether looking at private genes.

The identification of a large number of cancer-related genes for each patient also allows us to ask whether any of these genes are targetable with known drugs. We are able to identify for each patient several drugs that directly target genes (Fig 6B). Many of these drugs are or have been in clinical trials or are widely used for current cancer therapy as indicated in Fig 6B in bold. Notably, preferential allele expression in aurora kinase genes (AURKA) is significant in two patients. AURKA are targets of new drugs currently undergoing clinical trials [41].

Looking further at networks of drug targetable genes (Fig 6C) we observe that significant genes in our analysis form an interconnected network and that drugs exist that can target different points in this network. Targeting these genes, which form central networks in cells, are likely to result in measurable effects on cancer cells.

**Fig 7:**
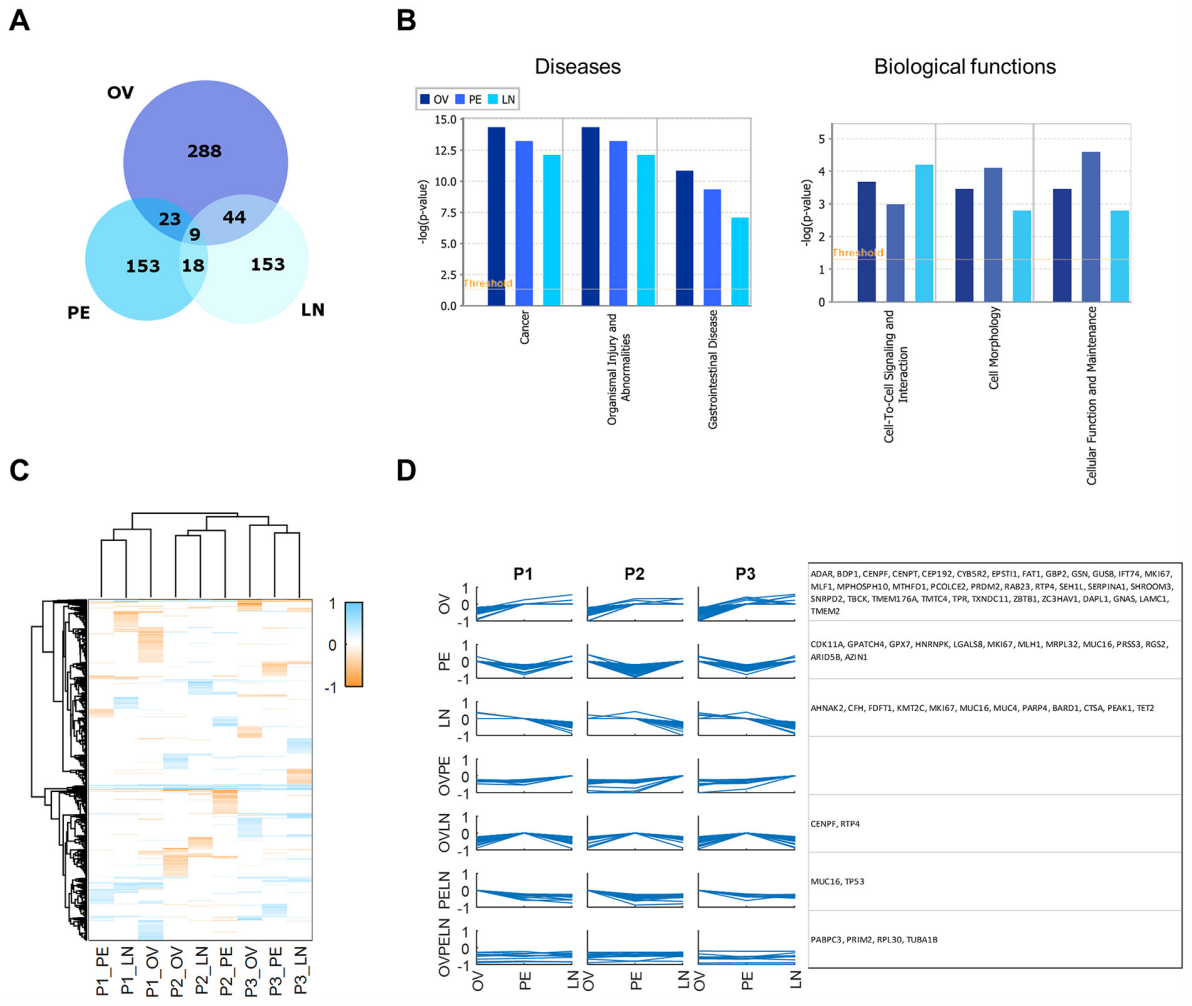
Site-specific preferential allele expression. A) Overlap of preferentially expressed variant alleles for the different sampled sites (OV, PE, LN). Numbers in intersecting or non-intersecting regions indicate the number of shared genes. B) Ingenuity pathway analysis of the different genes in each site showing the top 3 predicted diseases and the biological functions for each site. Note that gastrointestinal disease indicates cancer of the gastrointestinal system. Threshold (orange line) is set at a p-value of 0.05. C) Hierarchical clustering analysis of RNA-exome allele fraction differences expression differences show that different sites have largely different allelic expression patterns. As in previous figures, a positive allelic difference indicates a reference allele is preferentially expressed while a negative allelic difference indicates an alternate allele is preferentially expressed. Patients are also different with few shared alleles. D) Parallel plots of allele fraction differences for different patients and different site expression patterns. The site or combination of sites where there is a negative allele fraction difference is indicated by the site names (OV, PE, LN) for ovary, peritoneum and lymph node. Gene names that appear in at least two patients for a given pattern are indicated to the right of the parallel plot.

Finally, since our data includes primary and metastatic samples, we also analyzed the allele fraction differences at a site-specific level to identify any site-specific patterns. While there was little overlap between genes at the site level (Fig 7A), the disease pathways and biological functions were found to be the same in the different sites (Fig 7B). We then performed a hierarchical clustering analysis on significant allele fraction differences for all patients and sites (Fig 7C). As expected, patients clustered together but every sample and site clustered independently at the variant level. We also identified shared patterns across all possible clusters of negative allele fraction differences. These clusters represent sets of variants that show similar patterns of significant differential allele expression (Fig 7D). These clusters are the OV, PE, LN, OVPE, OVLN, PELN and OVPELN clusters. The PE, LN and PELN clusters are especially interesting as they are metastatic clusters. Interestingly, *TP53* appears in the PELN cluster indicating that metastasis samples (PE and LN) are overexpressing the variant TP53 more than in the ovary sample.

## Discussion

The keys results of our analysis are the identification of a large number of shared genes showing preferential variant allele expression among three different patients. We identify 28 shared genes many of which are known to be involved in ovarian cancer or other cancers and occur in cellular pathways that are relevant to tumor biology. These shared elements could form the basis for the biological similarities among ovarian cancers and lead to new therapeutic targets and better patient targeting of current therapies.

The observed preferential allele expression could indicate potential selection for that allele due to providing growth or survival benefits to the tumor. Alternative causes for significant preferential allele expression include artifacts of library preparation, alignment or sequencing. Preferential allele expression may also occur without biological or tumor significance. However, the evidence indicates that our analysis is capturing a biologically relevant set of genes. First, the number of variants that we identify as being significant is a small proportion of the total variants (Fig 4A and 4B) which indicates that there is no large systematic issue. Second, we validated the exome read ratios with SNP6 array data (where possible) and find excellent agreement between the array and exome data (S6 Fig). Third, we identify a large number of shared genes between different patients. Within these shared genes there are many variant types indicating that there is no bias for specific variant types. Some variants are the same in all patients while some variants are different in two or three other patients. Fourth, many of the genes we identify have very well known or plausible effects on tumor biology. Fifth, as discussed previously and below, many of the genes we identify with significant allele fraction differences are known drug targets. Sixth, comparing cell line data to patient data reveals large differences in the type of variants observed. There is also overall large similarity within patients than within cell lines suggesting that the preferentially expressed variant alleles in patients are specific. We therefore conclude that while there could be some biases due to low coverage or a higher error rate in one dataset, the majority of genes we identify are relevant to tumor biology and merit further investigation.

There are multiple mechanisms that can give rise to preferential allele expression. Copy number changes, including loss of an allele (LOH, loss of heterozygosity), RNA editing and allele specific methylation are three such mechanisms. However, our analysis methods that consider both the genomic and transcriptomic content would likely not detect preferential allele expression solely caused by copy number changes as we subtract the RNA read fraction from the DNA read fraction. For RNA editing, while our analysis would detect novel RNA only alleles, we restrict in this work our analysis to alleles that occur in both the exome and RNA sequencing data. This is because additional evidence would be needed to be have confidence in the novel RNA alleles. Allele specific methylation [42] could explain the preferential allele expression in our data as one allele could be methylated therefore reducing the expression of that allele relative to the unmethylated allele. Future experiments combining genome, transcriptome and methylation sequencing could determine the number and identity of preferentially expressed alleles modulated by methylation.

Our filtering procedure was stringent but it is possible that some of our data is due to different types of technical errors. Improving this analysis further could involve excluding reads from error-prone regions, applying more robust statistical methods and validating the expression data with site-specific qPCR. In addition, while we focused in this study on non-synonymous mutations or other high impact mutations, analyzing the synonymous mutations could lead to additional insight into tumor biology as synonymous mutations have been shown recently to act as drivers in tumors [43].

Another interesting subset of genes in our data are genes affecting cell adhesion and migration. The set of 28 shared and significant genes includes *LAMC2* [33, 34], MMP7 [44] and TNC [45]. We have previously described a large enrichment in somatic mutations in adhesion genes using the TCGA ovarian cancer data [46] while others have identified cell adhesion gene enrichment in a multidimensional study of ovarian cancer data [47]. Cell adhesion and migration are essential processes in tumor development and metastasis and it would be interesting to further investigate the functional significance of the variants we identify.

One notable advantage of applying CGTS to tumor samples is to be able to identify significant preferentially expressed germline variants. Somatic mutation analysis and standard gene expression studies cannot investigate germline variation. Germline variants are the vast majority of variants in cells and understanding their role in tumor biology could be critical to designing effective therapies. Another advantage of CGTS is that there is no requirement for large numbers of patients---it is possible to identify significant preferential allele expression in one patient.

Aside from potentially uncovering cancer drivers and suggesting patient-specific therapies, the observation that there is significant overlap between patients in the genes that have preferentially expressed alleles suggests that there is less heterogeneity at this level than seen with somatic mutation analysis. This increased homogeneity at the allelic expression level could impact the clustering of patients and has therapeutic implications as more patients can be treated with the same drug.

Overall, site analysis seems to indicate that different sites while having different genes utilize similar pathways. Perhaps the differences between primary and metastasis sites are due to gene expression changes or post-translation modifications. These results, while puzzling, are quite interesting. Indeed, the previous two studies on multiple site sampling [4, 5] demonstrated a high degree of mutational heterogeneity but most patients have a good initial response at all sites to chemotherapy potentially indicating shared biological features despite mutational heterogeneity.

Understanding tumor biology to impact clinical care requires the integration of multiple datasets in clinically meaningful ways. This work adds an additional novel dimension to the large body of work in tumor biology by analyzing preferential allele expression using a combined genome and transcriptome sequencing approach. The striking result of finding a set of shared biologically relevant genes with preferentially expressed variant alleles suggests that these genes may be driver genes. Future work characterizing these genetic variants individually and determining mechanisms of preferential allele expression would lead to greater understanding of the functional significance of these variants in tumor biology.

## Methods

### Ethics Statement

All samples were collected at the department of Gynecologic Oncology at the Institut Claudius Regaud. The project was reviewed and approved by the Institut Claudius Regaud Human research Ethics Committee. All patients included in the study gave informed written consent prior to surgery.

### Sample Collection

Three patients with Stage 3C high grade serous ovarian cancer were recruited for this study before any treatment at the Institut Claudius Regaud, Toulouse. All displayed metastasis in the lymph node and peritoneum. During primary cytoreductive surgery, tissue samples from the ovary, peritoneum and lymph node were collected and snap frozen. Biopsies were macrodissected and snap frozen sections were controlled and samples with 80% of tumor cells were selected. DNA and RNA was extracted with Qiagen RNA/DNA extraction kit. Additional clinical details are in S1 Text. Patients and sites are abbreviated (P1, P2, P3 for patients 1, 2, 3 and OV, PE, LN for ovary, peritoneum and lymph node).

Ovarian cancer cell line SKOV3 (HTB-77) was purchased from ATCC, primary ovarian cancer cell line GOC2 propagated in-house and human fibroblasts were maintained in culture (DMEM high glucose [Hyclone, Thermo Scientific], 10% FBS [Hyclone, Thermo Scientific], 1% Penicillin-Streptomycin-Amphotericyn B solution [Sigma], 1X Non Essential Amino-Acid [Hyclone, Thermo Scientific]). Human primary ovarian epithelial cells from ScienCell were cultured in poly-L-lysine-coated culture vessel (2 μg/cm2, T-75 flask) following ScienCell recommendations (Ovarian Epithelial Cell Medium (OEpiCM, Cat. No. 7311), 1% Ovarian Epithelial Cell Growth Supplement (OEpiCGS, Cat. No.7352), 1% penicillin/streptomycin solution (p/s, Cat.No 0503). Cultures were incubated in humidified 5% CO2 incubators and the media was replaced every 3 days. RNA and DNA were isolated using Qiagen Allprep DNA/RNA miniprep kit Cat. No. 80204 following manufacturer instructions and stored at -80 degrees Celsius before sequencing. Cells lines are abbreviated SK (SKOV3), GC (GOC2), FB (Fibroblast) and OE (ovarian epithelial).

### Sequencing and Alignment

RNA and exome sequencing was performed at Weill Cornell Medical College—Qatar, Genomics core. Exome capture was done using Agilent’s 38 mB SureSelect Human All Exon kit (patient tumor samples) and SureSelectXT2 Human All Exon V5 (cell lines and normal patient samples). Paired end, 100bp sequencing was done on an Illumina Genome Analyzer IIx (patient tumor samples) and Illumina HiSeq 2500 (cell lines and normal patient samples). Reads were aligned using BWA (version 0.7.9a) [48], indexed with samtools (version 0.1.18) [49], processed with Picard mark duplicates (http://picard.sourceforge.net, version 1.110) and realigned with GATK (version 3.1.1) [50, 51]. GATK bundle 2.8 was used for the reference genome hg19 and the realignment data. RNA was processed with Nugen Ovation v2 kit (patient tumor samples) and Nugen Ovation Single Cell RNA-Seq System (cell lines and normal patient samples) and 100bp sequencing was done on an Illumina HiSeq 2000 (patient tumor samples) and Illumina HiSeq 2500 (cell lines and normal patient samples). Resulting reads were aligned using RNA Star (version 2.4.0g1) in 2-pass mode [52] to the reference genome hg19 (S1 Fig) and deduplicated with Picard mark duplicates (version 1.110). Single nucleotide variants with mapping quality >20 and base quality > 20 were called using VARSCAN (version 2.3.7) [53, 54] from samtools mpileup output [55] combining all deduplicated and filtered exome and RNA samples and then annotated with SNPEFF (version 3.6) to obtain a VCF. Alignment and post processing read counts are shown in S1 Fig. The annotated VCF was then analyzed further with custom scripts.

### Allele Fraction Differences

Allele fractions were calculated for single nucleotide variants identified in exome and RNA sequencing data. We limited our analysis to reads with base and mapping quality greater than or equal to 20 and non-duplicated reads for both exome and RNA sequencing data. We further focused only on protein coding variants. Allele fractions are defined as the ratio of the number of reads of the reference allele to the total number of reads at a site. The Fisher Exact Test with the mid-P correction was used to obtain p-values between the RNA and Exome data (significance was set at a p-value less than 0.1). In addition, we imposed a filter that there should be at least eight reads for both the exome and RNA sequencing data to account for errors in sequencing and alignment [56]. The allele fraction difference (RAD) is calculated as the RNA allele fraction minus the exome allele fraction. Allele fraction differences range from -1 to 1 with negative differences meaning that the variant allele is preferentially expressed and positive differences meaning that the reference allele is preferentially expressed. Significant alleles refer to those alleles with significant negative preferential allele expression. Significant genes are those which have at least one variant allele has statistically significant preferential allele expression. Where normal sample (non-tumor) data is not available, we consider an allele to be likely germline if it also exists in dbSNP.

### Variant Annotation

SNPeff [57] was used to annotate the variants to identify protein coding variants. We use the same 'IMPACT' categorization as SNPEFF (High, Moderate, Low and Modifier). We used Ingenuity Pathway Analysis software (Ingenuity Systems) to annotate and functionally characterize gene lists and to identify drug targets. To identify SNPs that were in COSMIC [58], we downloaded the COSMIC complete export list (v72) and cross-referenced it with our variants based on genomic coordinates.

### Shared Gene Analysis

Identifying shared genes between groups is important as shared genes could point to common mechanisms. However, in sets where the number of elements is limited such as gene sets, it is possible to have shared genes that are likely to occur due to chance. To determine in our data if the number of shared genes or variants are significant, we calculated the number of shared variants from 1000 random selections of variants in our variant data (Shared Random) and compared that to the number of observed shared variants (Shared Observed). We then calculate a pseudo p-value which is the number of times the shared random selection exceeds or equals the shared observed number divided by the total number of random trials. A value of 0 means that in no random selection trial was the shared number of genes greater than the observed number of shared genes. A value of 1 means that in every random selection trial the number of shared genes was greater than or equal to the observed shared gene number. Custom Matlab code was developed for this analysis.

### Network Toplogy and Reactome Functional Interactions

Shared genes in both the patient positive and negative RAD sets were input into Jepetto [38], a Cytoscape interface for TopoGA [37] and topology calculated using the large dataset settings. Functional interactions were determined by using the Reactome Functional Interactions [39, 40] Cytoscape plugin using the 2014 dataset and using linker genes.

### Non-synonymous Variant Enrichment Analysis

This analysis counted at a gene level the frequency of non-synonymous and synonymous variants for different RAD sets: positive RAD, negative RAD and no RAD. For this analysis to be possible, genes must have more than one variant. Data was analyzed at numbers of variants greater than or equal to 2 and greater than or equal to 4. Custom Matlab code was used for this analysis.

### Gene Expression

RNA sequencing data was used to estimate relative gene expression. Alignments (see sequencing) were processed with FeatureCounts of Rsubread (version 1.18) [59] in paired-end mode, excluding overlapping reads and overlapping genes to map reads to genes (gencode v19 gene models). Filtration (remove all genes which have less than 0.1 counts per million) and normalization was done with edgeR (version 3.4.2) [60] to obtain reads per kilobase per million (rpkm) values for each gene. Patient and cell line data was analyzed identically.

### Visualization

GenomeBrowse (Golden Helix) was used to visualize the TP53 gene region. Cytoscape (version 3.2.1) [61] was used to visualize networks in Fig 5. Network diagrams in Fig 7 were made using Ingenuity software (Qiagen). Proportional Venn diagrams were made using BioVenn [62] and adjusted with Inkscape (version 0.91) Hierarchical clustering was done with built-in Matlab (version R2015a) (Mathworks) commands. Pileups, gene subplots, gene expression plots and parallel plots were made with custom Matlab scripts.

## Supporting Information

S1 Fig Exome and RNA sequencing read counts. (TIF)

S2 Fig Shows what are the significant changes when considering the control corrected differential allele ratios. The open colored circles are those significant differential allele expression before expression and the filled circles are the same after expression. Bottom table shows the numbers of the differences and these range from 4.5% to 9.4%.
(TIF)

S3 Fig Allele fraction difference histograms for different types of mutations for A) Patients and B) Cell lines. Patient, sites and cell line names are indicated on the left. The allele fraction difference on the x-axis ranges from -1 (only variant allele is expressed) to +1 (only normal allele is expressed). The frequency is indicated on the y-axis. Allele fraction differences less than -0.2 or greater than 0.2 were removed. Panels with horizontal lines have no variant of that type.
(TIF)

S4 Fig Cumulative density function plots of non-synonymous over synonymous ratios (non-syn/syn) for genes that have A) at least 2 significant variants or B) at least 4 significant variants. The grey, orange and blue lines are as indicated in the legend. Two notable observations are 1) no preferential allele expression always has low non-syn/syn ratios relative to at least one form of preferential allele expression and 2) there is a marked increase in non-syn/syn ratios in P1LN sample and 3) there are higher non-syn/syn ratios for +RAD genes.
(TIF)

S5 Fig Patient and cell line gene expression levels for the shared 28 genes. The x-axis of each panel shows the different sites (OV, PE, LN), the line colors correspond to different patients as indicated in the legend, and in letters (F, G, O, S) indicate the gene expression value for the cell line samples as indicated in the legend. The y-axis is the log2 (rpkm). Note the relative similarity of gene expression for most patients and sites. Exceptions to this similarity are ERRB2 which is high in patient 2 and MMP7 which shows marked changes in different sites.
(TIF)

S6 Fig Validation of Exome allele fractions with SNP6 data. Note that the exome allele fraction is based on the reference allele (as used in the manuscript) while the SNP6 frequency is based on the minor allele (as is customary in SNP array analysis). The red points are those where the minor/major allele needed to be switched as the minor allele matched the reference allele and the major allele matched the alternate allele. The green and black points represent sites where at least one of the RNA sequencing alleles did not match any of the SNP array alleles. The negative correlation is because we are comparing Exome Reference Allele Frequency to SNP array Minor Allele Frequency.
(TIF)

S1 Text Clinical description of patients. (DOCX)
