# Correction: Preferential Allele Expression Analysis Identifies Shared Germline and Somatic Driver Genes in Advanced Ovarian Cancer

**DOI:** 10.1371/journal.pgen.1005892

**Published:** 2016-02-18

**Authors:** Najeeb M. Halabi, Alejandra Martinez, Halema Al-Farsi, Eliane Mery, Laurence Puydenus, Pascal Pujol, Hanif G. Khalak, Cameron McLurcan, Gwenael Ferron, Denis Querleu, Iman Al-Azwani, Eman Al-Dous, Yasmin A. Mohamoud, Joel A. Malek, Arash Rafii

Information is missing from the Funding section. The correct funding information is as follows: This work was supported by the Qatar Foundation through the Qatar National Research Fund (grant programs NPRP4-640-1-096 and JSREP4-011-1-003) and the Qatar Science Leadership Program. The funders had no role in study design, data collection and analysis, decision to publish, or preparation of the manuscript.
