# Supplementary figures and images for: Preferential Allele Expression Analysis Identifies Shared Germline and Somatic Driver Genes in Advanced Ovarian Cancer
Source: PLoS Genet. 2016 Jan 6;12(1):e1005755. doi: 10.1371/journal.pgen.1005755 (PMC4703369; doi:10.1371/journal.pgen.1005755)

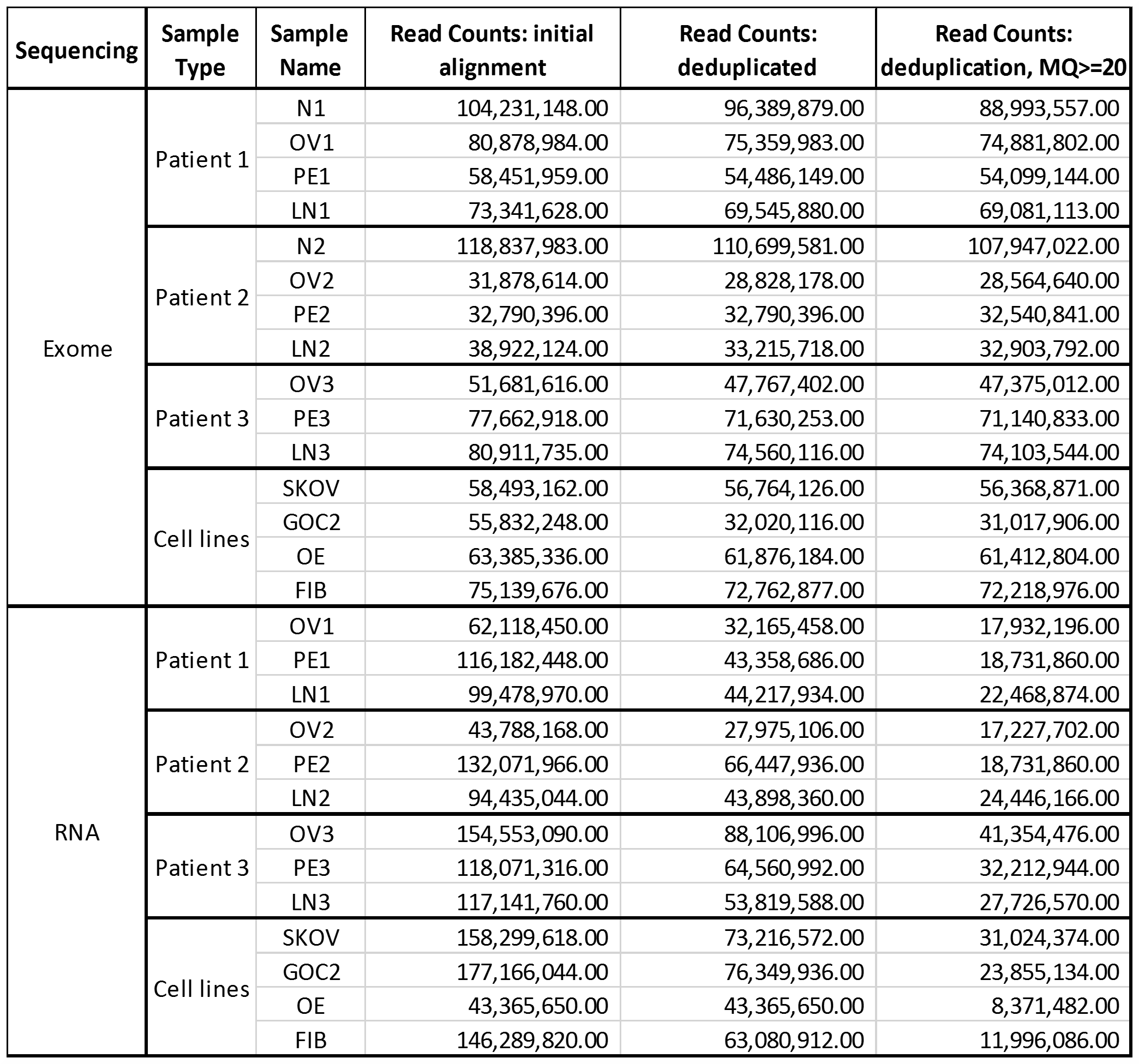

Supplement: S1 Fig — (TIF) [file pgen.1005755.s001.TIF]

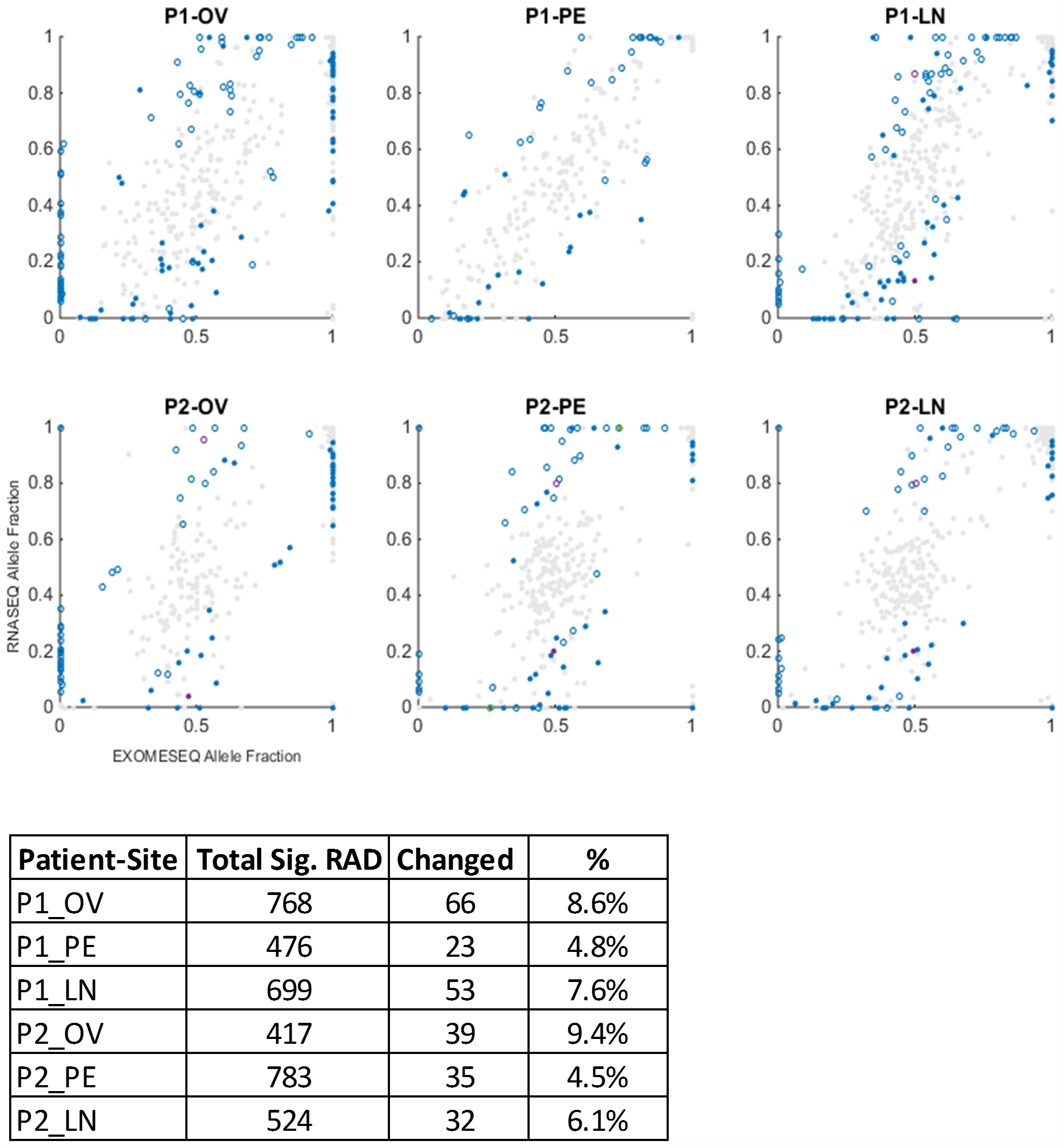

Supplement: S2 Fig — The open colored circles are those significant differential allele expression before expression and the filled circles are the same after expression. Bottom table shows the numbers of the differences and these range from 4.5% to 9.4%. (TIF) [file pgen.1005755.s002.TIF]

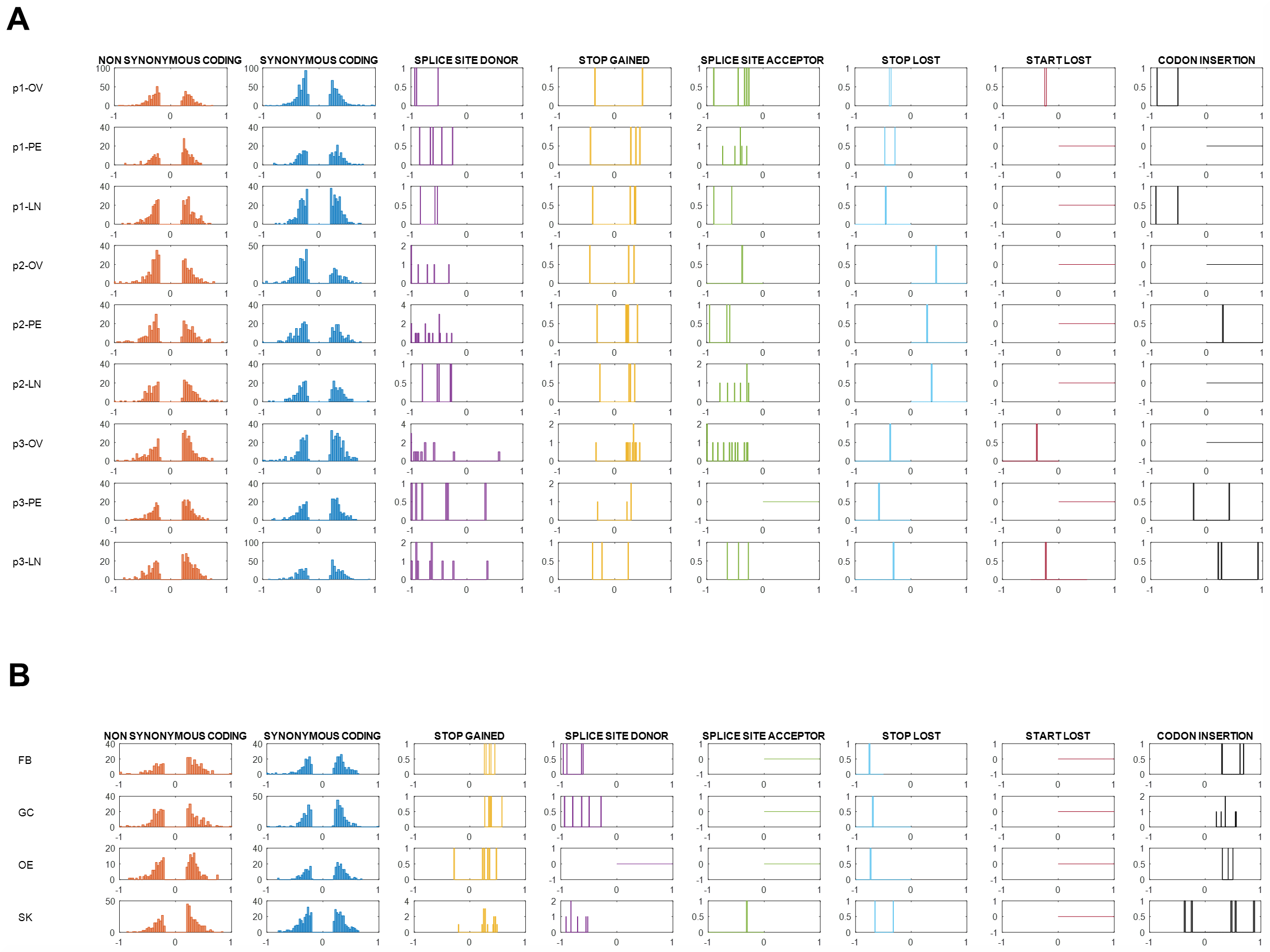

Supplement: S3 Fig — Patient, sites and cell line names are indicated on the left. The allele fraction difference on the x-axis ranges from -1 (only variant allele is expressed) to +1 (only normal allele is expressed). The frequency is indicated on the y-axis. Allele fraction differences less than -0.2 or greater than 0.2 were removed. Panels with horizontal lines have no variant of that type. (TIF) [file pgen.1005755.s003.TIF]

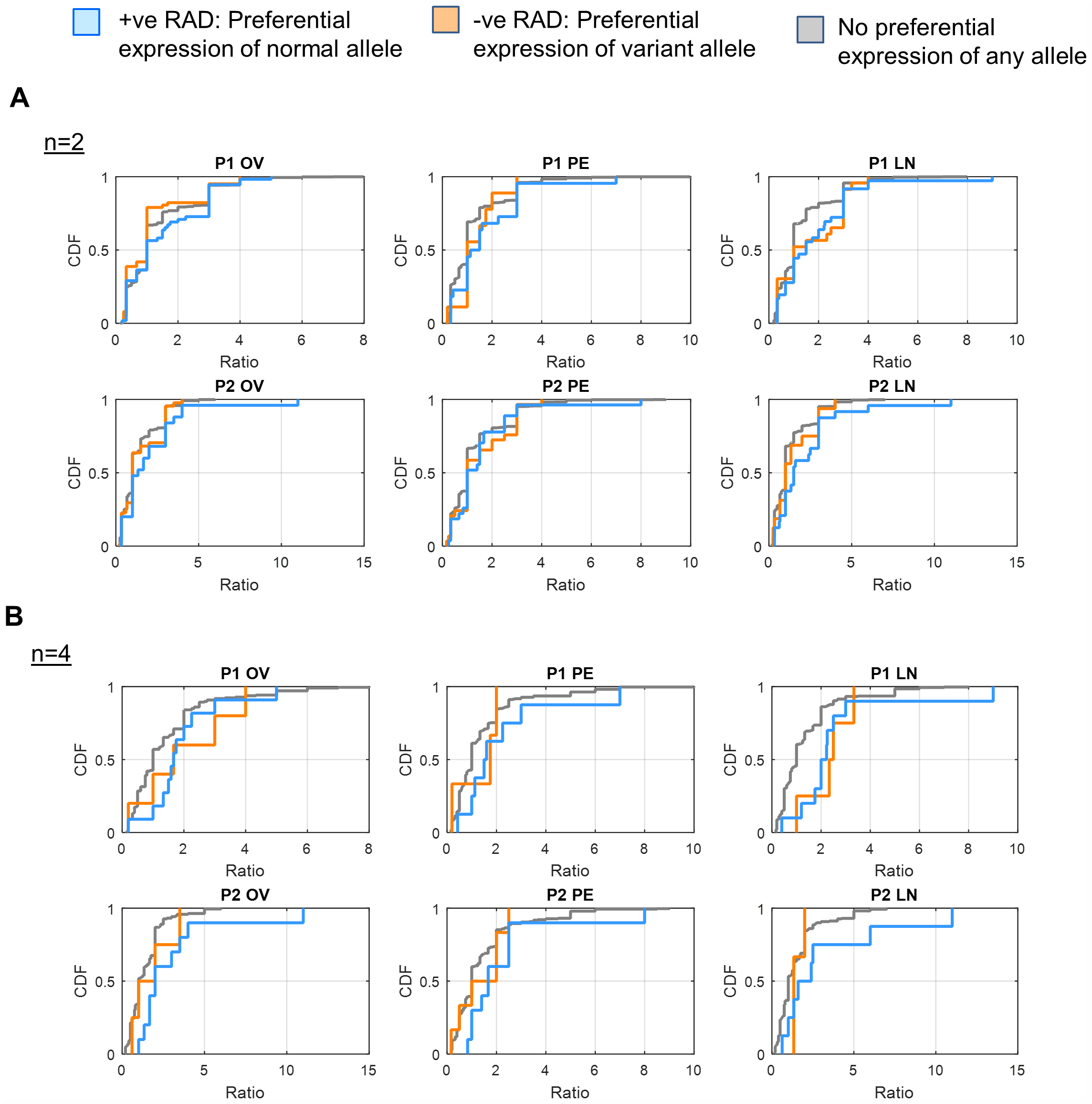

Supplement: S4 Fig — The grey, orange and blue lines are as indicated in the legend. Two notable observations are 1) no preferential allele expression always has low non-syn/syn ratios relative to at least one form of preferential allele expression and 2) there is a marked increase in non-syn/syn ratios in P1LN sample and 3) there are higher non-syn/syn ratios for +RAD genes. (TIF) [file pgen.1005755.s004.TIF]

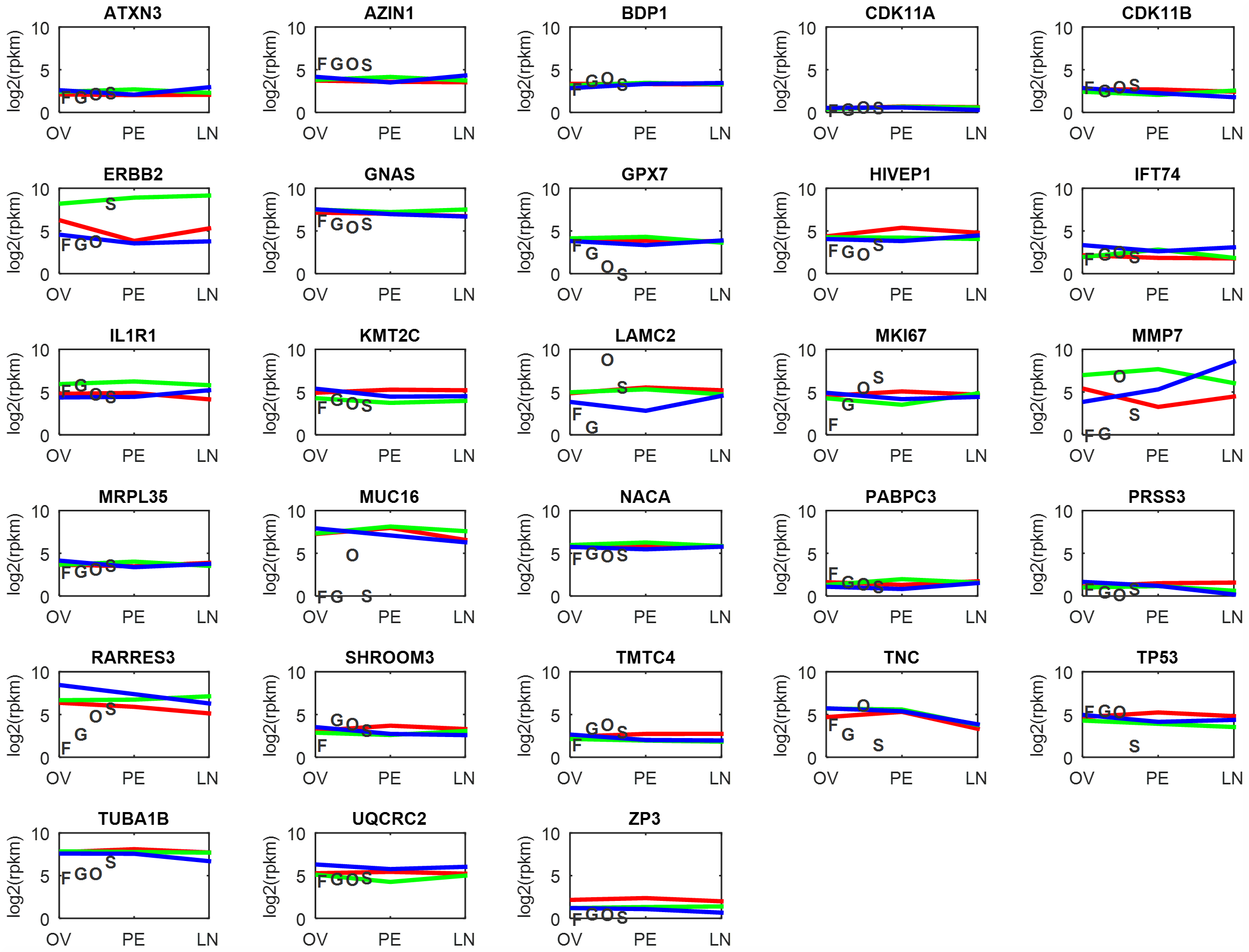

Supplement: S5 Fig — The x-axis of each panel shows the different sites (OV, PE, LN), the line colors correspond to different patients as indicated in the legend, and in letters (F, G, O, S) indicate the gene expression value for the cell line samples as indicated in the legend. The y-axis is the log2 (rpkm). Note the relative similarity of gene expression for most patients and sites. Exceptions to this similarity are ERRB2 which is high in patient 2 and MMP7 which shows marked changes in different sites. (TIF) [file pgen.1005755.s005.TIF]

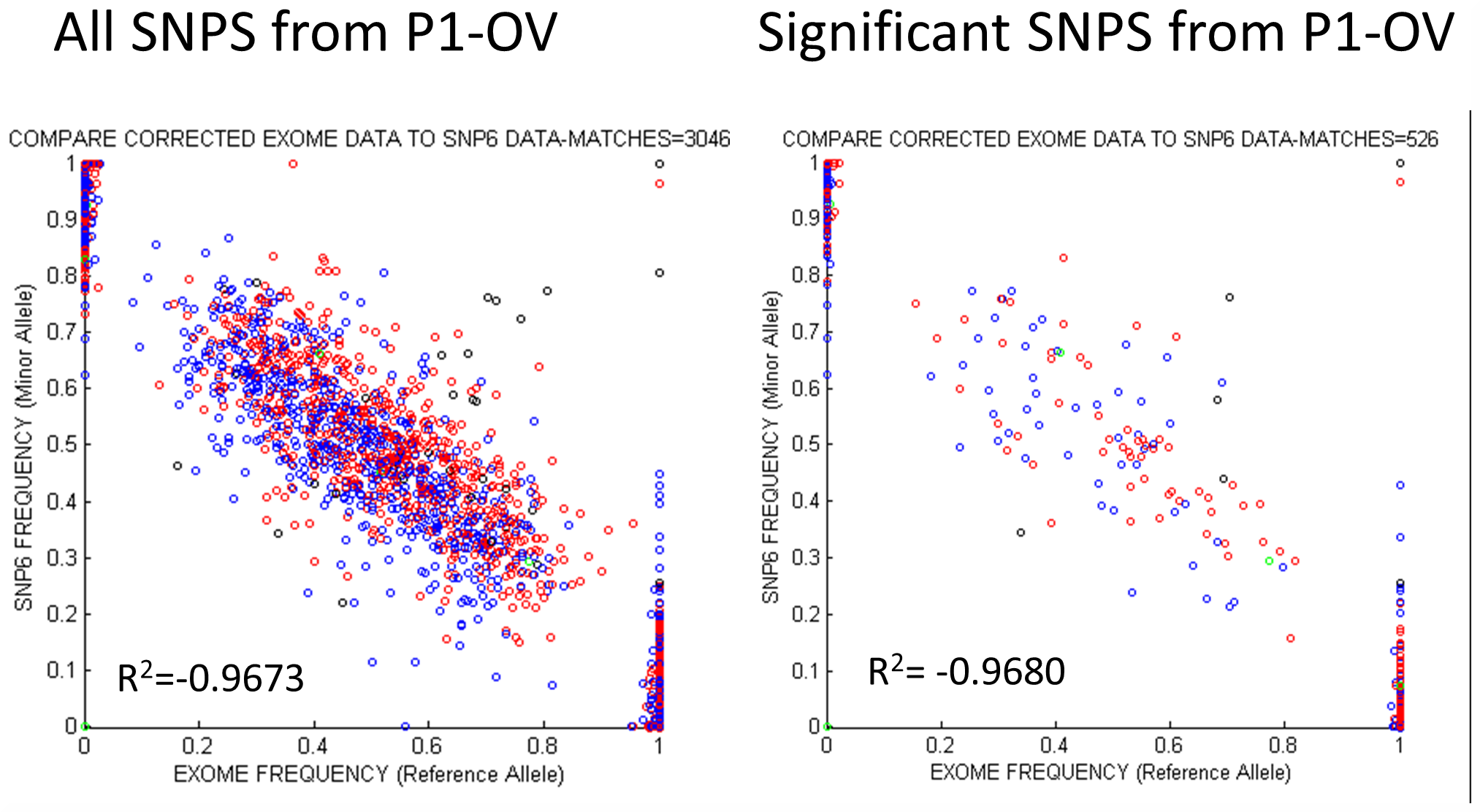

Supplement: S6 Fig — Note that the exome allele fraction is based on the reference allele (as used in the manuscript) while the SNP6 frequency is based on the minor allele (as is customary in SNP array analysis). The red points are those where the minor/major allele needed to be switched as the minor allele matched the reference allele and the major allele matched the alternate allele. The green and black points represent sites where at least one of the RNA sequencing alleles did not match any of the SNP array alleles. The negative correlation is because we are comparing Exome Reference Allele Frequency to SNP array Minor Allele Frequency. (TIF) [file pgen.1005755.s006.TIF]
