# Supplementary material for: Preferential Allele Expression Analysis Identifies Shared Germline and Somatic Driver Genes in Advanced Ovarian Cancer
Source: PLoS Genet. 2016 Jan 6;12(1):e1005755. doi: 10.1371/journal.pgen.1005755 (PMC4703369; doi:10.1371/journal.pgen.1005755)
Supplement: S1 Text — (DOCX) [file pgen.1005755.s007.docx]

**Clinical description for the patients:**

**Patient 1:**

Patient 1 underwent complete cytoreduction for limited peritoneal carcinomatosis to the upper abdomen and an important aortic lymph node involvement. She received adjuvant treatment with 6 cycles of carboplatin and taxol. She had isolated mediastinal nodal progression 15 months later treated by radiotherapy. Two years later she developed lung metastasis treated with Carboplatinum and Caelix. Disease progressed also in the abdominal cavity.

No BRCA mutation (test done) but estimated risk by BRCApro 89% and by Manchester 99%

Personal history: breast ca at 40years

Family hist: pat uncle prostate ca, pat uncle ORL ca, pat uncle esofaghus ca, pat grandfather prostate ca

AP: carcinomatous infiltration of the fallopian tube--> same aspect than the ovarian tumor

**Patient 2:**

Patient 2 presented with a symptomatic abdominal mass with péritonéal carcinomatosis. She underwent complete surgery  including upperabdominal procedures. Pathology exam found retroperitoneal lymph node involvement and difusse carcinomatosis corresponding to papillary serous and endometrioid carcinoma. She underwent adjuvant chemotherapy with Carboplatin and Taxol but progressed during chemotherapy with adominal disease and liver and lung metastasis. She died 3 months after a second line of chemotherapy was introduced due to thrombopenia associated to diffuse hemorrhage.

BRCA mutation unknown (test not done)

No personal history of ca

mother hepatic MTS from unknown primary tumor

AP: infiltration of the meso of the Fallopian tube, probably by peritoneal dissemination

**Patient 3:**

Patient 3 presented with small adnexal masses with extended retroperitoneal involvement but only minor peritoneal  carcinomatosis. She underwent complete cytoreduction and adjuvant chemotherapy with Carboplatinium and taxol. She progressed 15 months later with an isolated cerebellar lesion. She underwent neurosurgical resection and whole brain irradiation. 8 months later she had a second line of chemotherapy by carboplatinum and gemzar for retroperitoneal evolution. Disease progressed locally and she received a third line of chemotherapy.  She receives palliative care.

Stage IV at diagnosis

BRCA mutation unknown (BRCA test is done in France to <60 years or if personal-family history)

Family: pat aunt with breast ca

no personal history of previous ca

AP: no described invasion of the Fallopian tube

Note:

ca in the descriptions above is an abbreviation for cancer.
